# Treatment of malignant pleural effusion in non-small cell lung cancer with VEGF-directed therapy

**DOI:** 10.1080/07853890.2022.2071977

**Published:** 2022-05-11

**Authors:** Zhangqiang Xiang, Xiangyu Deng, Wenfeng He, Qian Yang, Laichao Ni, Marzieh Dehghan Shasaltaneh, Mazaher Maghsoudloo, Gang Yang, Jingbo Wu, Saber Imani, Qinglian Wen

**Affiliations:** a Department of Oncology, The Affiliated Hospital of Southwest Medical University, Luzhou, China; b Phase 1 Clinical Trial Center, Deyang People’s Hospital, Deyang, China; c Nuclear Medicine and Molecular Imaging Key Laboratory of Sichuan Province, Luzhou, China. The Affiliated Hospital of Southwest Medical University, Luzhou, China; d Department of Biology, Faculty of Science, University of Zanjan, Zanjan, Iran; e Laboratory of Systems Biology and Bioinformatics, Institute of Biochemistry and Biophysics, University of Tehran, Tehran, Iran; f Department of Genetics, Faculty of Advanced Science and Technology, Tehran Medical Sciences, Islamic Azad University, Tehran, Iran; g Department of Oncology, Anyue Hospital of Traditional Chinese Medicine, Second Ziyang Hospital of Traditional Chinese Medicine, Ziyang, China

**Keywords:** Malignant pleural effusion, non-small-cell lung cancer, vascular endothelial growth factor, bevacizumab, apatinib, cisplatin

## Abstract

**Background:**

Vascular endothelial growth factor (VEGF) is a critical regulator of malignant pleural effusion (MPE) in non-small-cell lung cancer (NSCLC). Bevacizumab (BEV) and apatinib (APA) are novel VEGF blockers that inhibit lung cancer cell proliferation and the development of pleural effusion.

**Methods:**

In this study, we established Lewis lung cancer (LLC) xenograft mouse models to compare the therapeutic effect of APA and BEV in combination with cisplatin (CDDP) against MPE. The anti-tumour and anti-angiogenic effects of this combination therapy were evaluated by ^18^F-FDG PET/CT imaging, TUNEL assay and Immunohistochemistry.

**Results:**

The triple drug combination significantly prolonged the overall survival of the tumour-bearing mice by reducing MPE and glucose metabolism and was more effective in lowering VEGF/soluble VEGFR-2 levels in the serum and pleural exudates compared to either of the monotherapies. Furthermore, CDDP + APA + BEV promoted *in vivo* apoptosis and decreased microvessel density.

**Conclusions:**

Mechanistically, LLC-induced MPE was inhibited by targeting the VEGF-MEK/ERK pathways. Further studies are needed to establish the synergistic therapeutic effect of these drugs in NSCLC patients with MPE.

**KEY MESSAGES**
Combined treatment of MPE with apatinib, bevacizumab and cisplatin can prolong the survival time of mice, reduce the content of MPE, decrease the SUV_max_ of thoracic tumour tissue, down-regulate the content of VEGF and sVEGFR-2 in serum and pleural fluid, and promote the apoptosis of tumour cells. Angiogenesis and MPE formation can be inhibited by down-regulation of HIF-1α, VEGF, VEGFR-2, MEK1 and MMP-2 molecular signalling pathway proteins.

## Introduction

Non-small-cell lung cancer (NSCLC) is the most prevalent among all lung malignancies, and accounts for most cancer-related deaths with an annual mortality rate of 1.6 million worldwide [1,2]. The first-line treatment against NSCLC includes surgery, chemo-immunotherapy or radiotherapy. Furthermore, several molecular therapies targeting immune checkpoints, epidermal growth factor receptor (EGFR), tyrosine kinases and cell cycle mediators have been developed in recent years, Nevertheless, the overall 5-year survival rate of NSCLC patients remains less than 14% [3]. The Lewis lung cancer (LLC) cell line was established from the lung tissues of C57BL/6 mice implanted with primary Lewis lung carcinoma cells. LLC is highly metastatic in immunocompetent mice and is routinely used to evaluate the efficacy of chemotherapeutic agents *in vivo* [4].

Malignant pleural effusion (MPE) is a common complication of advanced NSCLC that significantly lowers patient quality of life and shortens the overall survival (OS) [5]. It can be cleared by thoracentesis, intra-pleural drug infusion, biological response modifiers and drainage *via* simple puncture, although the final outcomes are usually unsatisfactory. The molecular mechanisms underlying the induction of MPE are complex and poorly understood. However, recent studies have implicated angiogenesis to be an important factor [6,7]. The metastatic pleural tumour cells infiltrate into lymphatic vessels and block reflux absorption of the pleural fluid, which triggers local inflammation in the pleura. The ensuing increase in the levels of vasoactive mediators such as vascular endothelial growth factor (VEGF), tumour necrosis factor (TNF) and chemokine ligand (CCL) 2, and the decrease in protective factors like endostatin increase the permeability of the pleural capillary walls, eventually leading to MPE [8]. Furthermore, the upregulation of oncogenic transcription factors in the pleural exudates exacerbates inflammation, vascular leakage, angiogenesis, tumour dissemination, and drug resistance. The VEGF family promotes endothelial cell proliferation and angiogenesis and is also associated with MPE in patients with advanced NSCLC [9,10]. Pre-clinical and clinical studies show that decreasing VEGF levels in NSCLC patients can significantly control pleural effusion [6,11,12]. Therefore, VEGF-targeted therapies are a viable palliative strategy for patients with MPE.

Bevacizumab (tradename: Avastin; BEV), a recombinant humanized monoclonal antibody targeting VEGF, was the first anti-angiogenic drug approved in the United States to treat MPE [13,14]. It binds to human VEGF (VEGF-A) and blocks the VEGF-VEGFR pathway, thereby inhibiting the growth of new blood vessels in the tumour and promoting tumour cell apoptosis. The tyrosine kinase inhibitor (TKI) apatinib (YN968D1, APA) selectively targets VEGFR-2 [15,16] and has been approved for treating advanced or metastatic chemo refractory gastric cancer in China [17,18]. APA can effectively block the kinase activity of VEGFR-2, c-kit and c-src, as well as the phosphorylation of VEGFR-2, c-kit and platelet-derived growth factor receptor beta (PDGFRb) [19]. It can inhibit the proliferation, migration and tubular formation of human umbilical vein endothelial cells *in vitro*, and the germination of aortic rings in rats [12,19–21]. In addition, APA has shown satisfactory efficacy against solid tumours with tolerable side effects [21–23]. Interestingly, the combination of APA with other chemotherapeutic drugs can effectively inhibit the growth of various human xenografts in animal models [19,24,25]. However, the effect of APA either alone or in combination with other anti-angiogenic drugs on MPE has not been reported so far.

VEGFR-1 and VEGFR-2 are the main receptors of VEGF that relay signals of cell proliferation, angiogenesis, and vascular permeability [26,27]. VEGFR-2-dependent endothelial cell proliferation in NSCLC tumours is directly correlated with MPE [28,29] and can be controlled by intrathoracic administration of cisplatin (CDDP). Several studies have reported beneficial outcomes of the combination of traditional chemotherapeutic drugs and anti-angiogenic drugs against solid tumours [30–34]. We recently reported the efficacy of the simultaneous angiogenesis blockade in NSCLC with BEV and APA [8], and the combination of BEV and CDPP is an effective and safe option for clearing MPE [35,36]. Several studies have shown that intrapleural anti-VEGF and/or anti-EGFR administration reduced pleural fluid volume and the levels of inflammatory mediators without affecting patient survival [37]. Furthermore, VEGFR-2 blockade alone is not sufficient to achieve the maximum therapeutic effect against MPE since tumour-derived VEGF can also promote metastasis *via* the VEGF/VEGFR-1 signalling pathway [38]. However, the therapeutic efficacy of anti-angiogenic drugs in combination with CDDP, and the underlying mechanisms, remain unclear [39,40]. To this end, we evaluated the effects of the triple combination of BEV, APA and CDDP on Lewis lung cancer (LLC) cell-induced MPE in a mouse model. The novel drug combination inhibited tumour angiogenesis and growth by targeting the VEGF/VEGFR-related pathways and can potentially improve the prognosis of NSCLC patients with MPE.

## Materials and methods

### Drugs and reagents

APA mesylate tablets (Aitan) were purchased from Hengrui Co. Ltd. (Lianyungang, Jiangsu, China), BEV from Roche Pharmaceutical Co. Ltd. (Shanghai, China), and CDDP from Jiangsu Hansoh Pharma Group Co. Ltd. (Lianyungang, Jiangsu, China). The optimum doses and timing for APA [19,41], BEV and CDDP [13,42] were determined based on previous reports and a preliminary experiment was as follows: CDDP − 2 mg/kg per day once a week, APA − 200 mg/kg per day daily, and BEV − 5 mg/kg twice a week.

### Cell culture

LLC cell line was purchased from Otwo Biotech (catalog number: HTX1771; Shenzhen Inc., Guangdong, China) and cultured in Dulbecco's Modified Eagle's medium (DMEM, HyClone, Thermo Scientific, USA) supplemented with 10% foetal bovine serum (Thermo Fisher Scientific, Waltham, MA, USA) and 1% (v/v) antibiotics (100 units/mL penicillin G sodium and 100 μg/mL streptomycin sulphate; Sigma-Aldrich, St Louis, MO, USA) under 5% CO_2_ at 37 °C. The cell line was fingerprinted within 6 months of the experiments [43].

### Establishment of LLC xenograft model of MPE and treatment regimen

In this study, we were established a MPE mouse model with LLC cell lines according to the standard model [43–45]. For more details, 98 male C57BL/6 mice (age 6–8 weeks) were purchased from Dashuo Animal Laboratory Centre (Chengdu, China) and housed in specific-pathogen-free (SPF) conditions at 21 ± 1 °C, 40 ± 10% relative humidity and a 12-h light/dark cycle, with *ad libitum* access to water and food. The MPE xenograft model was established by injecting 200 μL LLC cell suspension (1 × 10^6^ cells/mL) into the right thoracic cavity of each mouse anaesthetised with 1% pentobarbital (50 mg/kg) through the right axillary front and the sixth costal space [43]. Nine days later, the mice were intrathoracically injected with different combinations of BEV and CDDP [45] and given oral gavage of APA or intra-lumen injection of normal saline for eight consecutive days (*n* = 14 each, *n* = *n*1 + *n*2) [46]. The specific treatment groups were as follows: (1) Control – intra-lumen injection of 0.1 mL normal saline on day 10 and tube feeding with 0.5 mL from days 11 to 17, (2) CDDP − 2 mg/kg CDDP on day 10, (3) APA – normal saline on day 10 and 200 mg/kg APA from days 11 to 17, (4) BEV - normal saline on day 10 and 5 mg/kg BEV on days 11 and 15, (5) CDDP + APA group: 2 mg/kg CDDP on day 10 and 200 mg/kg APA from days 11 to 17, (6) CDDP + BEV group: 2 mg/kg CDDP on day 10 and 5 mg/kg BEV on days 11 and 17, and (7) CDDP + APA + BEV group: 2 mg/kg CDDP on day 10, 200 mg/kg APA from days 11 to 17, and 5 mg/kg BEV on days 11 and 15. Six mice were randomly selected from each group (n1) for overall survival analysis, and the remaining 8 (n2) were monitored twice a day for changes in body weight and euthanized on the day 19. The general conditions of the animals, including eating, activity, appearance, and response to external stimuli, were observed twice a day. We carried out euthanasia through excessive anaesthesia. The specific method was to closely observe the condition of the mice after intraperitoneal injection of pentobarbital (150 mg/kg). When the voluntary breathing disappeared, the voluntary heartbeat was suspended, and the eyeball pupil was fixed and dilated, the mice were determined to be dead. On day 19, mice (n2) were euthanized under anaesthesia. After euthanasia, all samples, blood, pleural exudates and tumour tissues, were collected, and the volume of bilateral pleural effusion was measured. The tumour-bearing mice were dissected and the gross findings were photoed. Three days after the detection of tumour growth, all the remaining mice underwent PET/CT to observe the MPE formation and calculate the rate of MPE formation at this time point; The observation was repeated on the 18th day, and the rates of MPE formation were also calculated. As previously described [8], the pleural effusion in the bilateral thoracic cavity was gently extracted using a syringe and the fluid volume was recorded. All procedures were conducted as per the guidelines of the National Institutes of Health Guide for the Care and Use of Laboratory Animals (NIH Publications No. 8023, revised 1978), and approved by the Institutional Animal Care and Treatment Committee of Southwest Medical University, Luzhou, Sichuan, China.

### ^18^f-FDG micro-positron emission tomography (PET)/computed tomography (CT) imaging

The metabolic status of the tumour tissues was analysed on day 18 in the n2 group by ^18^F-FDG micro-PET/CT using the Inveon micro PET/CT animal scanner (Siemens, Munich, Germany). Briefly, the mice were fasted for at least 8 h before the scan and anaesthetized by an intraperitoneal injection of 1% pentobarbital at the dose of 50 mg/kg [47]. Fifteen minutes later, the animals were checked for depth of anaesthesia by a toe pinch to verify no response and then were injected with ^18^F-FDG. Each mouse was then injected with 0.1–0.2 mL of 150–200 μCi ^18^F-FDG through the tail vein and placed in the centre of the PET/CT ring field of view 30–40 min later. After induction of anaesthesia, mice were placed in a supine position, with their heads fixed [47]. Whole body scans were performed in the 2 D mode with 10 min per bed position. Other scanning parameters were 80 kV, 500 μA and 1.5 mm slice collimation. The tumour region of interest (ROI) in the chest cavity of each mouse was delineated, and the maximum standardized uptake value (SUV_max_) was calculated using the single hottest pixel within the tumour. The PET/CT images and data were analysed by two nuclear medicine experts.

### Immunohistochemistry

The tumour nodules were fixed in 10% neutral formaldehyde, embedded in paraffin, and cut into 5-μm-thick sections. For immunohistochemical analysis, the tumour sections were dewaxed and soaked in 0.01 M sodium citrate (pH 6) for 24 h, and heated in a pressure cooker heated for 90 s. After blocking non-specific reactions with 2% normal mouse serum (Santa Cruz Biotechnology, Santa Cruz, CA) for 30 min at room temperature (RT), the sections were incubated in levamisole (Vector Laboratories, Burlingame, CA) to quench endogenous alkaline phosphatase. The slides were then rinsed with PBS, and incubated overnight with rabbit anti-hypoxia-inducible factor (HIF)-1α (1:100, Bioss Biotechnology Co. Ltd., Beijing, China), anti-VEGF (1:200, Abcam Trading Co. Ltd., Shanghai, China), anti-VEGFR-2 (1:200, Abcam Trading Co. Ltd., Shanghai, China), anti-MEK1 (1:300, Abcam Trading Co. Ltd., Shanghai, China), anti-cluster of differentiation 31 (CD31) (1:200, Abcam Trading Co. Ltd., Shanghai, China), and anti- matrix metalloproteinase (MMP)-2 (1:100, Proteintech Group Inc., Wuhan, China) primary antibodies at 4 °C. Normal anti-rabbit IgG, normal pre-immune anti-goat IgG, or tris-buffered saline were used as negative controls. After washing once, the sections were sequentially probed with biotinylated goat anti-rabbit IgG and horseradish peroxidase-labelled anti-goat secondary antibody (1:200, Aspen medical products Co. Ltd., California, USA) for 50 min at RT, rinsed four times with PBS, and incubated with DAB substrate (ZLI-9033, ZSGB Biotechnology Co. Ltd., Beijing, China) until the desired colour was achieved (Table 1). Subsequently, the sections were counterstained with haematoxylin, dehydrated through an ethanol gradient and xylene, and fixed with neutral glue. All sections were visualized under the Axioplan 2 microscope (Zeiss, Toronto, ON, Canada) and the images were analysed using Zeiss Axiovision software (Zeiss; New York, NY, USA). Five random, non-overlapping fields per section were observed at 100× and 400× magnification using a MicroPublisher imaging system (Q-IMAGING, Surrey, BC, Canada), and the immuno-positive areas were evaluated by two independent pathologists. All sections were counterstained in haematoxylin nuclear fast red for 10 min. The expression intensities of the different proteins were calculated as a/*b* × 100% × c [48], where a, b and c respectively indicate the positively stained area, total area and average grey level of each section at 400× magnification. The microvessel density (MVD) was calculated as the mean area occupied by microvessels in five CD31^+^ hotspots at 100× magnification. In addition, the number of microvessels was counted in these areas at 400×, and the average was calculated [49]. The images were separately analysed by two pathologists.

**Table 1.** Detailed profiles of used antibodies.

Antibody name	Supplier	Clone	Catalogue No.	Dilutions	Incubation Time/Temp.
CD31	Abcam	Rabbit polyclonal	ab28364	1:200	**Overnight at 4 °C**
HIF-1α	Bioss	Rabbit polyclonal	bs-0737R	1:100	**Overnight at 4 °C**
VEGF	Abcam	Rabbit polyclonal	ab52917	1:200	**Overnight at 4 °C**
VEGFR-2	Abcam	Rabbit polyclonal	ab194806	1:200	**Overnight at 4 °C**
MEK1	Abcam	Rabbit polyclonal	ab178876	1:300	**Overnight at 4 °C**
MMP-2	Proteintech Group	Rabbit polyclonal	10373-2-AP	1:100	**Overnight at 4 °C**
HRP labelled goat Anti-rabbit IgG	Aspen	Goat anti-rabbit IgG	AS-1107	1:200	60 min at 37 °C

CD31: cluster of differentiation 31; HIF-1α: hypoxia-inducible factor-1α; HRP: horseradish peroxidase; MEK1: MAPK/ERK kinase 1; MMP-2: matrix metalloproteinase 2; VEGF: vascular endothelial growth factor; VEGFR-2: vascular endothelial growth factor receptor 2.

### Enzyme-linked immunosorbent assay (ELISA)

The levels of soluble VEGFR-2 (sVEGFR-2) and VEGF in the blood and pleural exudates were evaluated using specific sandwich ELISA kits according to the manufacturer’s instructions (Wuhan, Boster Biological Technology, China). The absorbance [optical density (OD) value] of each well at 450 nm was measured within 10 min of adding the stop solution provided in the kit, and the values were normalized to that of the blank. A standard curve was then plotted according to the concentration of the standards and the corresponding OD values, and the amount of sVEGFR-2 and VEGF was calculated using a regression equation. The lower limit of detection for sVEGFR-2 was 300 ng/L and that for VEGF was 12.5 ng/mL. The ELISA reader and washer used in this study were Stat-Fax 2100 and Stat-Fax 2600 (USA), respectively.

### Terminal deoxynucleotidyl transferase dUTP nick end labelling (TUNEL) assay

Pleural tumour tissues were stained using a TUNEL kit (Roche Applied Science, Indianapolis, IN, USA) according to the manufacturer’s protocol, and imaged and analysed using Image J in a blinded manner. The TUNEL-positive apoptotic cells were counted in at least five random fields (magnification: 200×), and the relative percentage was calculated. The experiment was repeated thrice.

### Statistical analysis

All statistical analyses were performed using SPSS software version 23.0 (SPSS Inc., Chicago, IL, USA) and GraphPad Prism software version 7.0 (GraphPad Software Inc., La Jolla, CA, USA). All tests were repeated three times or more. Quantitative data are expressed as mean ± standard deviation. Multiple groups were compared by one-way analysis of variance (ANOVA), and the average number of pairwise comparisons was determined by Tukey’s test. Spearman’s rank correlation coefficient test was used to determine the association between two variables. Survival duration and rate were evaluated by the Kaplan–Meier method, and the log-rank test was used to compare survival curves. For all tests, two-sided *p values* less than .05 were considered statistically significant.

## Results

### CDDP + APA + BEV prolonged survival and decreased LLC growth and pleural effusion

**Figure 1.**
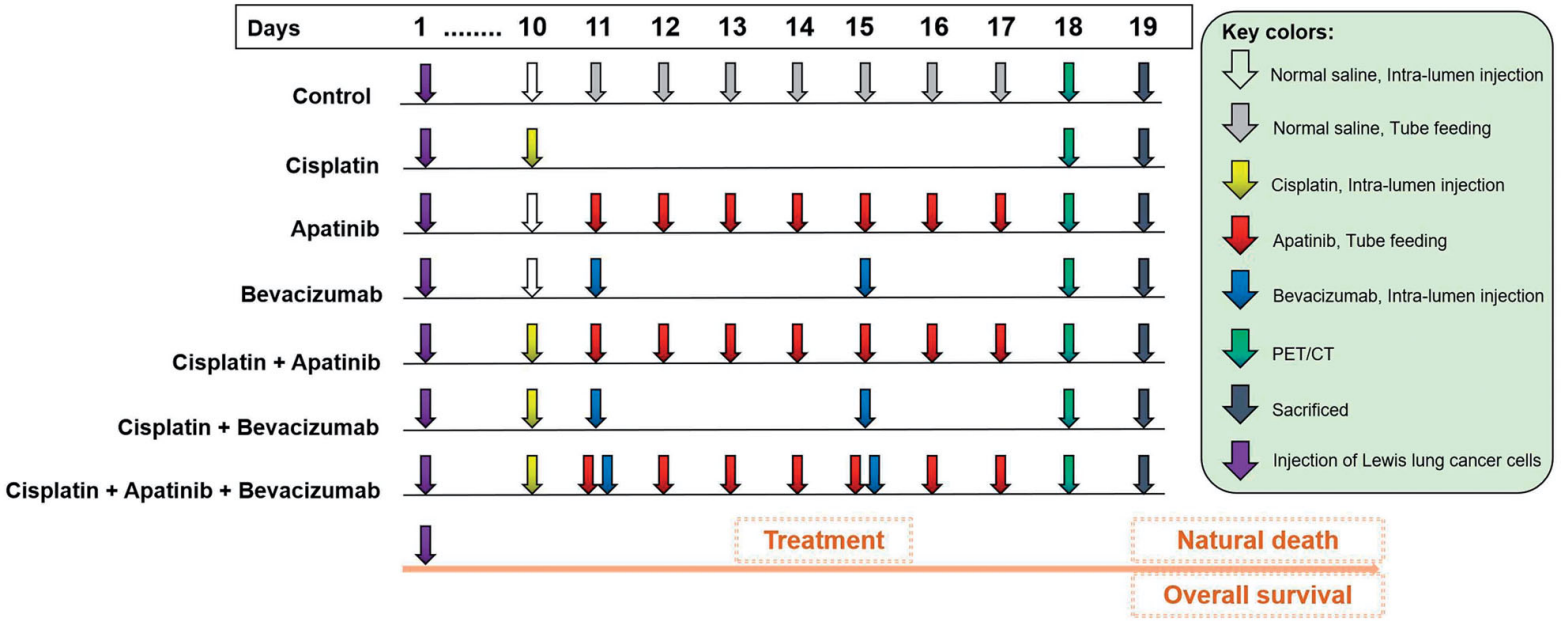
BEV, APA and CDDP treatment regimen. Each arrow corresponds to the individual drug. APA: apatinib; BEV: bevacizumab; CDDP: cisplatin; PET/CT: Fluorine-18-fluorode oxyglucose (^18^F-FDG) micro-positron emission tomography/computed tomography.

The effects of BEV, CDDP, APA and their different combinations were tested in an LLC-induced MPE model. The grouping, therapeutic regimens and subsequent analyses are outlined in Figure 1.

**Figure 2.**
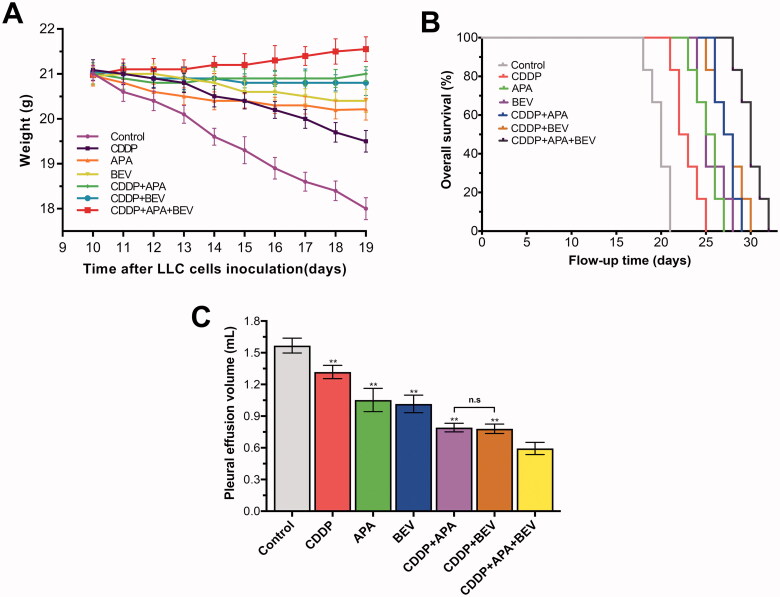
Combination therapy decreased MPE and prolonged survival of tumour-bearing mice. (**A**) The body weight of tumour-bearing mice in the indicated treatment groups. The data are shown as the mean ± SD. (**B**) Kaplan–Meier survival curves for the differentially treated mice. (**C**) Bilateral pleural effusion volume in the differentially treated mice. Data are expressed as mean ± SD. ** *p <* .01 vs CDDP + APA + BEV. APA: apatinib; BEV: bevacizumab; CDDP: cisplatin; LLC: Lewis lung cancer; n.s.: not significant; SD: standard deviation.

As shown in Figure 2(A), on day 19, the body weight of the mice treated with BEV (20.40 ± 0.26 g), APA (20.21 ± 0.24 g) or CDDP (19.50 ± 0.24 g) alone was significantly higher than that of the mice treated with normal saline (Control: 18.00 ± 0.24 g; *p* < .01). Combining CDDP with either APA (21.00 ± 0.16 g) or BEV (20.80 ± 0.28 g) further improved the body weight of tumour-bearing mice (*p* < .01 compared to control), although no significant difference was observed between the CDDP + APA and CDDP + BEV groups (*p* = .67). The highest average body weight was observed in the CDDP + APA + BEV group (21.53 ± 0.28 g). Consistent with this finding, the drug triad also significantly prolonged the median survival of tumour-bearing mice to 30 days compared with only 20 days observed in the control group (Figure 2(B)). Furthermore, APA (25 days), BEV (25 days) and CDDP (22 days) monotherapies also prolonged survival (*p* < .01 compared to Control), although the individual drugs were less effective compared to their combination (*p* < .01). Finally, pleural effusion volume was also significantly lower in the CDDP + APA + BEV group (0.59 ± 0.06 mL) compared with that in the CDDP + BEV (0.78 ± 0.04 mL), CDDP + APA (0.79 ± 0.04 mL), APA (1.05 ± 0.11 mL), BEV (1.01 ± 0.08 mL) and CDDP (1.32 ± 0.06 mL) groups. CDDP + APA and CDDP + BEV had a similar effect on MPE as that observed with body weight (*p* = 1.00). Taken together, the combination of BEV, APA and CDDP significantly decreased tumour growth and pleural effusion and prolonged the survival of the tumour-bearing mice compared to the individual drugs.

### The triple combination therapy reduced glucose metabolism in LLC xenografts

**Figure 3.**
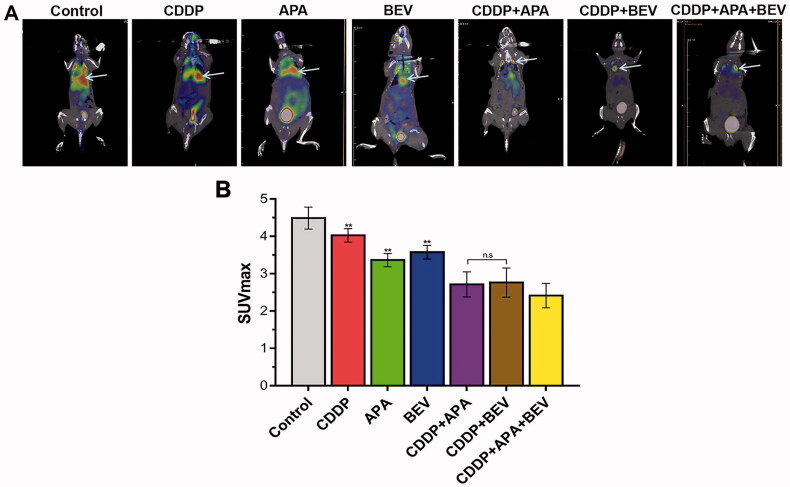
Combination therapy reduced glucose metabolism in LLC xenografts. (**A**) Representative ^18^F-FDG PET images of the indicated groups on day 18. (**B**) SUV_max_ values in the indicated treatment groups. The arrow shows the location of the internal tumours. Data are expressed as mean ± SD. ** *p* < .01 vs CDDP + APA + BEV. ^18^F-FDG PET/CT: Fluorine-18-fluorode oxyglucose (^18^F-FDG) micro-positron emission tomography/computed tomography; APA: apatinib; BEV: bevacizumab; CDDP: cisplatin; SD: standard deviation; LLC: Lewis lung cancer; n.s.: not significant; SUVmax: maximum standardised uptake value.

The metabolic effects of different therapies were evaluated by ^18^F-FDG PET/CT (Figure 3) with SUV_max_ as the quantitative index for radioactive uptake [50]. The SUV_max_ values for BEV (3.58 ± 0.18), CDDP (4.03 ± 0.18) and APA (3.36 ± 0.17) monotherapies were significantly lower than that for the control group (4.49 ± 0.29). The different combinations of APA, BEV and CDDP further lowered the SUV_max_ values, with the lowest was observed for CDDP + APA + BEV (2.41 ± 0.33). No significant differences were observed between the CDDP + APA and CDDP + BEV groups (2.71 ± 0.34 vs 2.76 ± 0.39; *p* = 1.00). Taken together, the combination therapy inhibited the growth of tumour nodules in the pleural cavity by effectively reducing glucose metabolism.

### The triple combination therapy inhibited angiogenesis in tumour tissues

**Figure 4.**
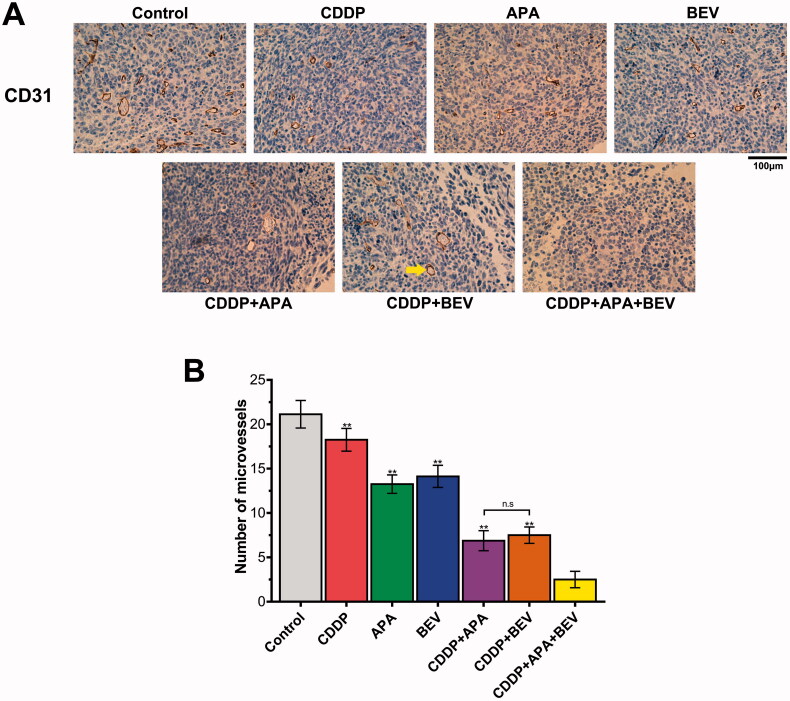
Combination therapy suppressed LLC tumour angiogenesis *in vivo*. (**A**) Representative images showing CD31 immunostaining in the tumour tissues of the differentially treated mice (original magnification, ×400). The yellow arrow indicates positively stained areas. (**B**) MVD in the pleural tumour nodules of the indicated groups. Data are expressed as mean ± SD. ** *p* <.01 vs CDDP + APA + BEV. APA: apatinib; BEV: bevacizumab; CDDP: cisplatin; CD31: cluster of differentiation 31; LLC: Lewis lung cancer; MVD: microvessel density; n.s.: not significant.

Tumour growth and angiogenesis were also evaluated in terms of the MVD. As shown in Figure 4(A), the combination therapy significantly decreased the intensity of CD31^+^ staining in the tumour tissues compared to the monotherapies and untreated control. Consistent with this, the MVD in the pleural tumour nodules was significantly decreased in the CDDP + APA + BEV (2.80 ± 0.83), CDDP + BEV (7.60 ± 0.87) and CDDP + APA (7.04 ± 1.12) groups compared to that in each of the monotherapy groups and the control group (21.13 ± 1.55). The MVD was highest in the untreated controls, followed by the CDDP, BEV, APA, CDDP + BEV, CDDP + APA and CDDP + APA + BEV groups in that order. Taken together, BEV and APA in combination with CDDP synergistically inhibited tumour angiogenesis, resulting in significant tumour regression.

### The combination of APA or BEV with CDDP synergistically induced apoptosis of tumour cells

**Figure 5.**
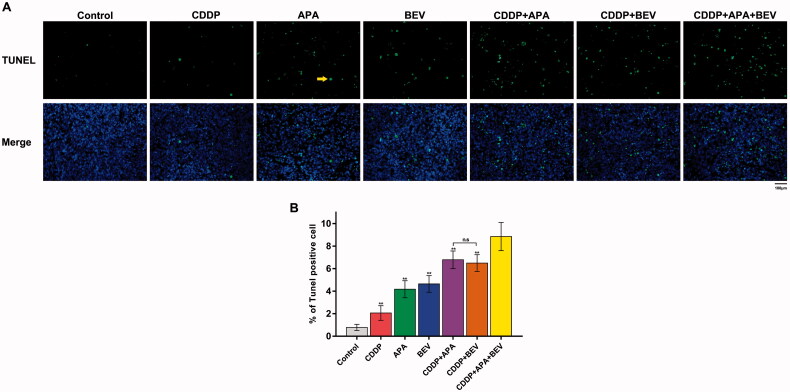
Combination of bevacizumab, apatinib and cisplatin increased apoptosis in mouse xenografts. (**A**) Representative images of TUNEL-stained (green) sections counterstained with DAPI (blue). Original magnification, ×200. (**B**) Percentage of TUNEL-positive cells in the pleural tumour nodules. ** *p <* .01 vs CDDP + APA + BEV. APA: apatinib; BEV: bevacizumab; CDDP: cisplatin; n.s.: not significant; TUNEL: terminal deoxynucleotidyl transferase dUTP nick end labelling.

As shown in Figure 5, the apoptotic index of pleural tumour nodules was markedly higher in the CDDP + APA + BEV group compared with that in other groups. The percentage of TUNEL^+^ apoptotic tumour cells was highest in mice treated with the drug triad, followed by CDDP + APA, CDDP + BEV, BEV, APA and CDDP in that order (Figure 5(A)) and were significantly higher compared that in the untreated controls (*p* < .01). Furthermore, the apoptosis rate was significantly higher in the CDDP + APA + BEV group compared to the rest (8.85 ± 1.23%, *p* < .01 vs. all). The combination of APA or BEV with CDDP increased apoptosis compared to either monotherapy (*p* < 0.01). Therefore, the combination of an angiogenesis blocker with CDDP can synergistically induce apoptosis in the tumour cells.

### The drug triad downregulated VEGF/sVEGFR-2 level in tumour-bearing mice

**Figure 6.**
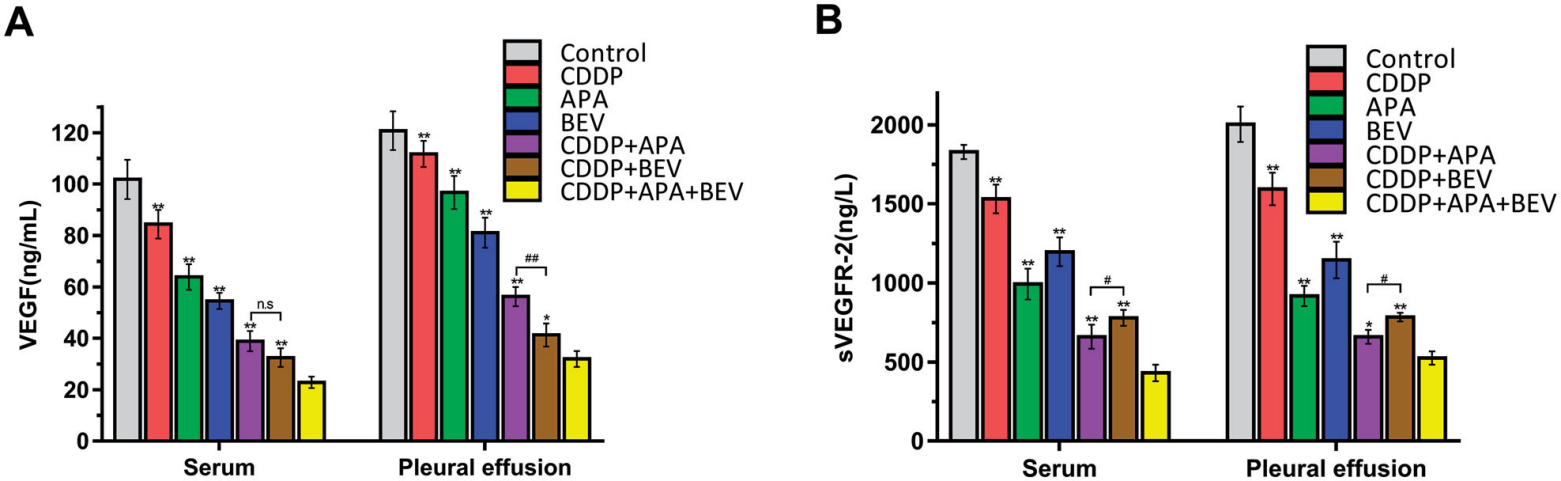
Combination therapy synergistically down-regulated VEGF/sVEGFR-2. Bar graphs showing VEGF (**A**) and sVEGFR-2 (**B**) levels in the serum and pleural exudates of the differentially treated mice. * *p* < .05, ** *p* < .01 vs CDDP + APA + BEV; ^#^ *p* < .05, ^##^ *p* < .01 vs CDDP + BEV. APA: apatinib; BEV: bevacizumab; CDDP: cisplatin; n.s.: not significant; sVEGFR-2: soluble vascular endothelial growth factor receptor 2; VEGF, vascular endothelial growth factor.

To gain further insights into the mechanisms underlying the anti-angiogenic effects of BEV, APA and CDDP, we next analysed the levels of VEGF and sVEGFR-2 in the serum and pleural fluid of the tumour-bearing mice. As shown in Figure 6(A), VEGF levels in the serum (22.85 ± 2.20 ng/mL; *p* < .01 vs. all) and pleural exudates (31.96 ± 3.04 ng/mL; *p* < .05 vs. all) of mice in the CDDP + APA + BEV group were the lowest compared to that in other treatments groups. Consistent with this, the level of sVEGFR-2 was the highest in the untreated control group in the serum and pleural fluid, followed by the CDDP, BEV, APA, CDDP + BEV, CDDP + APA and CDDP + APA + BEV groups in that order. There was a significant difference between other groups with CDDP + APA + BEV (*p* < .05). Taken together, CDDP + APA + BEV achieved simultaneous inhibition of VEGF and sVEGFR-2 in the serum and pleural fluid, which decreased neo-angiogenesis and growth.

### The triple drug combination therapy synergistically inhibited the VEGF/MAPK/ERK pathway in pleural tumour nodules

**Figure 7.**
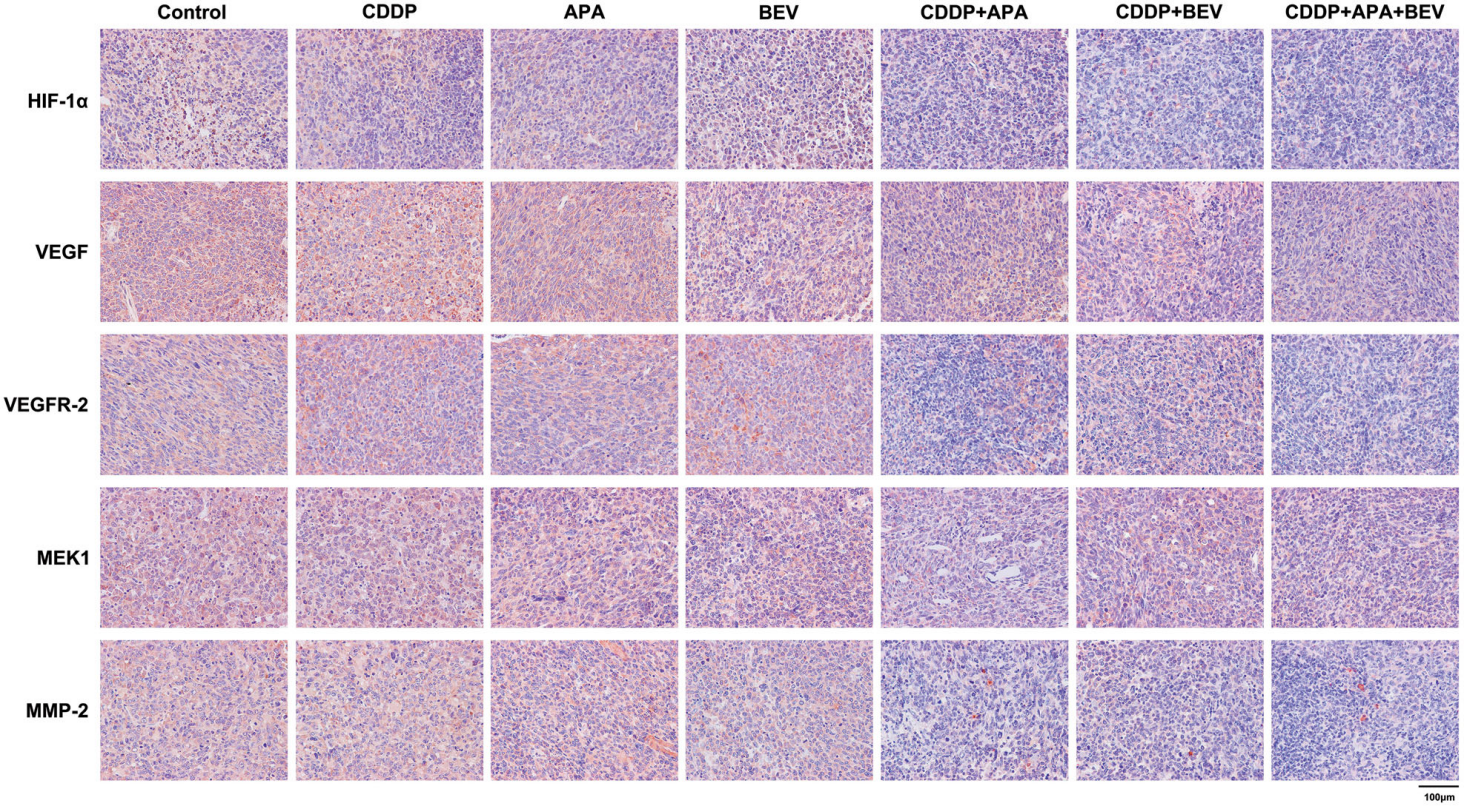
Representative images showing immunostaining for HIF-1α, VEGF, VEGFR-2, MEK1 and MMP-2 in the tumour tissues of the indicated groups (original magnification, ×400). APA: apatinib; BEV: bevacizumab; CDDP: cisplatin; HIF-1α: hypoxia-inducible factor-1α; MEK1: MAPK/ERK kinase 1; MMP-2: matrix metalloproteinase 2; VEGF: vascular endothelial growth factor; VEGFR-2: vascular endothelial growth factor receptor 2.

**Figure 8.**
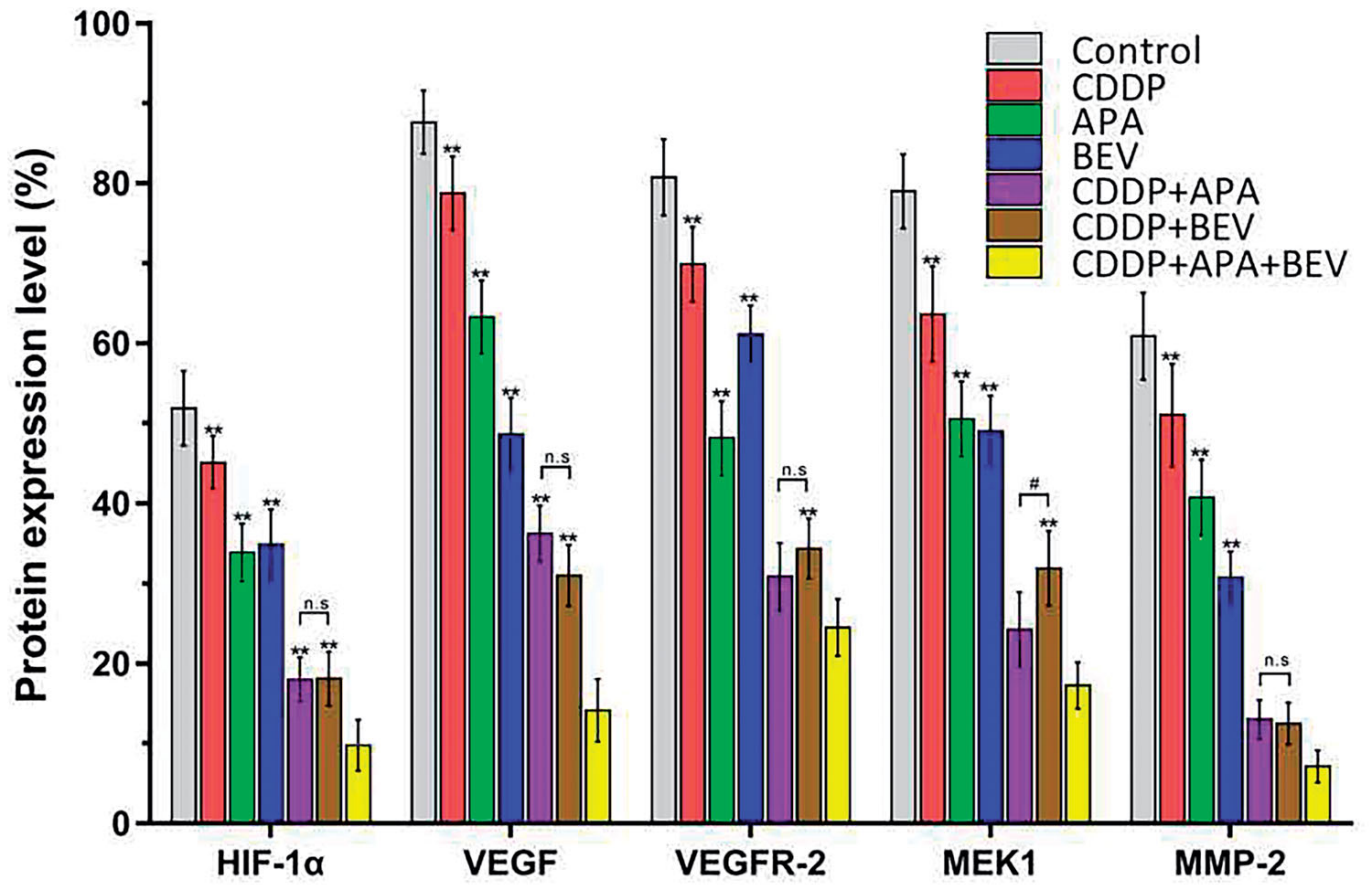
Combination therapy targets the VEGF-induced MAPK/ERK signalling pathway in tumour nodules. Horizontal bars show mean ± SD of cells positive for HIF-1α, VEGF, VEGFR-2, MEK1 and MMP-2. ** *p* < .01 vs CDDP + APA + BEV; ^#^ *p* < .05 vs CDDP + BEV. APA: apatinib; BEV: bevacizumab; CDDP: cisplatin; HIF-1α: hypoxia-inducible factor-1α; MEK1: MAPK/ERK kinase 1; MMP-2: matrix metalloproteinase 2; n.s: not significant; SD: standard deviation; VEGF: vascular endothelial growth factor; VEGFR-2: vascular endothelial growth factor receptor 2.

The MAPK pathway is a critical oncogenic pathway and the target of multiple chemotherapeutic drugs [51]. Therefore, we next analysed the expression levels of the downstream VEGF-induced mitogen-activated protein kinase kinase (MEK)/extracellular signal-regulated kinase (ERK) signalling pathway. As shown in Figures 7 and 8, the triple drug combination therapy synergistically inhibited the *in situ* levels of HIF-1α, VEGF, VEGFR-2, MEK1 and MMP-2 proteins compared the control group (*p* < .01), as well as the monotherapy groups (*p* < .01). Interestingly, HIF-1α, VEGF, VEGFR-2 and MMP-2 expression was similar between the CDDP + APA and CDDP + BEV groups (*p* > .05). These findings indicate that BEV, APA and CDDP inhibited angiogenesis in LLC xenografts by blocking the MAPK/ERK and Ras/Raf/MEK/ERK pathways.

## Discussion

The aim of our study was to analyse the possible synergistic effect of the combination of BEV, APA and CDDP against NSCLC complicated with MPE. The combination therapy induced apoptosis in LLC cells and reduced the volume of pleural effusion in tumour-bearing mice, which significantly prolonged their survival. Mechanistically, the drug triad suppressed NSCLC growth and angiogenesis by inhibiting the VEGF-induced MAPK/ERK signalling pathway. This is the first study to identify the therapeutic value of combining BEV, APA and CDDP in NSCLC.

VEGF plays a vital role in promoting angiogenesis and the pleural invasion of VEGF-overexpressing tumour cells initiates MPE formation [52]. At present, MPE clearance mainly relies on intra-pleural administration of antineoplastic agents, thoracentesis, pleurodesis, and the use of an indwelling pleural catheter (IPC) [53,54]. Although the exact mechanism of MPE formation is unclear, there is evidence implicating direct invasion of tumour cells into the pleura, tumour-induced blockade of blood and lymphatic capillaries, obstruction of lymphatic circulation by lymph node metastasis, and increased permeability of pleural capillary walls induced by tumour-secreted cytokines. Studies show that VEGF-dependent angiogenesis and increased capillary permeability are the main factors inducing MPE [55]. VEGF promotes vascular leakage by opening the intercellular junctions and creating a fenestration on the endothelial lining [55], and its levels are significantly higher in malignant as opposed to benign pleural exudates [56]. VEGFR-1 and VEGFR-2 overexpression also aggravate MPE and tumour angiogenesis *via* autophosphorylation and endothelial cell signal transduction [57,58]. Thus, reducing VEGF levels can effectively control pleural effusion. BEV and APA are novel angiogenesis blockers that respectively target VEGF-A and VEGFR-2 [17,59]. Studies increasingly show that an anti-angiogenic drug combination can significantly reduce MPE and recurrent ascites formation by targeting VEGFR-2 [29,60]. Furthermore, intrapleural injection of the platinum-based CDDP is routinely used to kill tumour cells and control MPE since the drug cannot cross the pleural barrier easily and therefore accumulates over time to therapeutically high concentrations. However, the combination of BEV and CDDP can clear MPE with an 80% effectivity rate compared to 47.8% of CDDP alone [36].

The decrease in MPE relieves the squeezing of lungs and reduces severe hypoxia, which increases food intake in mice [8,61,62]. Previous studies have shown that the drug combination can inhibit the growth of transplanted tumours in mouse models without significant weight loss and toxicity, and prolong survival [63,64]. In our study as well, combining CDDP with either APA or BEV maintained the body weight of tumour-bearing mice, which can be attributed to the higher food intake compared to the untreated control animals. Tumour growth inhibition is accompanied by reduced ^18^F-FDG uptake, which is indicative of lower glucose metabolism and better prognosis *in vivo* [65–67]. Studies have correlated a lower SUV_max_ of tumour masses to slower tumour growth and a better clinical outcome [68,69]. We found that the different drug combinations significantly reduced ^18^F-FDG uptake by the tumours compared to the monotherapies.

Soluble VEGFR-2 (sVEGFR2) contains the extracellular domain of the VEGFR-2 receptor but lacks the tyrosine kinase domain and is activated in the hypoxic tumour microenvironment [70,71]. W. Xu *et al.* [72] found that the plasma sVEGFR-2 levels in renal cancer patients decreased significantly following surgical resection and adjuvant treatment with VEGFR-targeting TKIs. Consistent with this, the combination treatment significantly decreased VEGF and sVEGFR-2 levels in the serum and pleural exudates of LLC xenograft-bearing mice, which likely translates to decreased neo-angiogenesis, normalised tumour blood vessels, and a more balanced tumour microenvironment.

**Figure 9.**
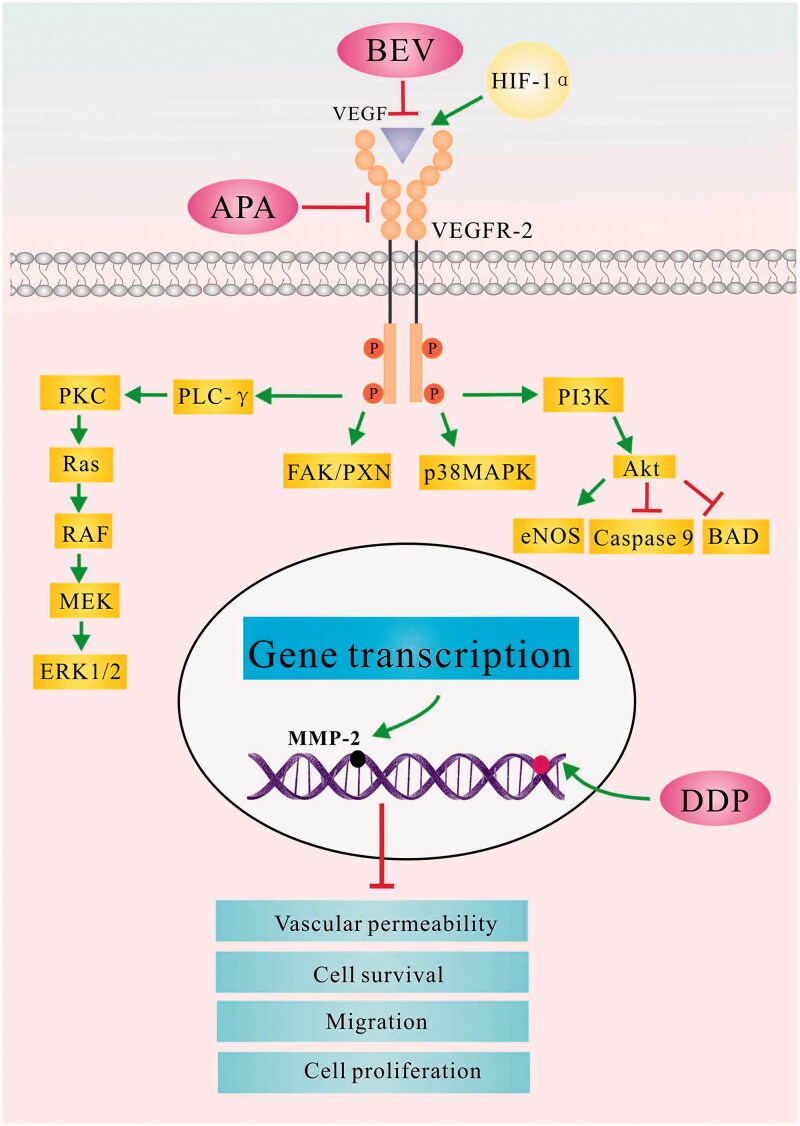
The possible mechanism underlying the therapeutic effect of bevacizumab, apatinib and cisplatin in the LLC tumour model. APA: apatinib; BAD: BCL2 associated agonist of cell death; BEV: bevacizumab; CDDP: cisplatin; eNOS: encodes endothelial nitric oxide synthase; FAK/PXN: focal adhesion kinase (FAK)/paxillin pathway; HIF-1α: hypoxia-inducible factor-1α; LLC: Lewis lung cancer; MAPK: mitogen-activated protein kinase; MEK: mitogen-activated protein kinase; MMP-2: matrix metalloproteinase 2; PI3K: phosphatidylinositol 3-kinases; PLC- γ: phospholipase C gene- γ; PKC: protein kinase C; RAS: intrinsic GTPase RAS; RAF: Raf proto-oncogene, serine/threonine kinase; VEGF: vascular endothelial growth factor; VEGFR-2: vascular endothelial growth factor receptor 2.

The combination of BEV, APA and CDDP inhibited neo-angiogenesis in the LLC xenografts by blocking the MAPK/ERK and Ras/Raf/MEK/ERK pathways, and had a more pronounced effect on HIF-1α and MMP-2 than either drug alone. HIF-1 is the key activator of hypoxia-induced angiogenesis and is regulated by the tumour suppressor gene von Hippel Lindau (VHL). Under normoxic conditions, HIF-1α is rapidly degraded by the ubiquitin-proteasome pathway under the control of VHL. However, hypoxia or VHL deletions/mutations results in the heterodimerization of HIF-1α and HIF-1β, which then translocates to the nucleus and activates VEGF transcription [73]. MMP-2 is an oncogenic protein that degrades extracellular matrix proteins and triggers mediates matrix remodelling and vascularization [74]. It lies downstream of the VEGF signalling cascade and plays an important role in the invasion and metastasis of tumour cells. Studies show that binding of VEGF with VEGFR-2 receptor induces dimerization of the latter, which promotes tumour angiogenesis by activating the C-RAF-MEK-MAPK pathway [75,76]. VEGF/VEGFR-2 interaction also increases microvascular permeability, and the proliferation, invasion, migration and survival of endothelial cells [77,78]. As shown in Figure 9, CDDP, APA and BEV simultaneously target different intermediates in the VEGF-induced MAPK/ERK signalling pathway and NRF2-dependent antioxidant response pathways, which enhances the anti-tumour effect.

The present study has some limitations that ought to be considered. The small sample size and short study duration may have affected the results. In addition, we did not analyse the molecular and cellular mechanisms underlying the synergistic action of BEV, APA and CDDP. Further studies are needed to determine the long-term and short-term toxicity profiles of these drugs, as well as the mechanistic basis of their action. Some *in vitro* studies have shown that inhibition of VEGF signalling effectively stimulates anti-tumour immunity and enhances the efficacy of TKIs and immune checkpoint blockade [76,79,80]. For example, the combination of BEV + Atezolizumab achieved a promising objective response ratio in renal cell carcinoma [81]. Simultaneous blockade of PD-L1 and VEGF can suppress formation of blood and lymphatic vessels in the tumours, and promote immune cell adhesion, trafficking and activation [82]. At the same time, the study also needs further protein/mRNA expression analysis to corroborate the results of immunohistochemistry. Furthermore, other possible drug combinations, such as anti-PD-L1 and anti-VEGF should also be tested in NSCLC. Therefore, clinical studies with larger cohorts are needed to fully investigate the therapeutic efficacy of BEV, APA and CDDP in NSCLC patients.

In summary, the combination of BEV, APA and CDDP prolonged survival of LLC tumour-bearing mice by reducing MPE and glucose metabolism. A better understanding of this combination will define new approaches to enhance drug efficacy in the treatment of NSCLC patients with MPE by possible moderation of VEGF/MAPK/ERK pathways.

## Data Availability

All the datasets generated and analysed during the present study are available from the corresponding author on reasonable request.

## Abbreviations

APA: Apatinib
BEV: Bevacizumab
CD31: Cluster of differentiation 31
CDDP: Cisplatin
ERK: Extracellular signal-regulated kinase
ELISA: Enzyme-linked immunosorbent assay
^18^F-FDG: ^18^F-fluorodeoxyglucose
HIF-1α: Hypoxia-inducible factor-1α
IPC: Indwelling pleural catheter
LLC: Lewis lung cancer
MPE: Malignant pleural effusion
MEK: Mitogen-activated protein kinase kinase
MEK1: MAPK/ERK kinase 1
MMP-2: Matrix metalloproteinase 2
MVD: Microvessel density
OS: Overall survival
NSCLC: Non-small cell lung cancer
PlGF: Placenta growth factor
PET/CT: Positron emission computed tomography
ROI: Region of interest
SPF: Specific pathogen free
sVEGFR-2: Soluble vascular endothelial growth factor receptor 2
SUV_max_: Maximum standard uptake value
TKI: Tyrosine kinase inhibitor
VEGF: Vascular endothelial growth factor
